# Preparation of RGD Peptide/Folate Acid Double-Targeted Mesoporous Silica Nanoparticles and Its Application in Human Breast Cancer MCF-7 Cells

**DOI:** 10.3389/fphar.2020.00898

**Published:** 2020-06-16

**Authors:** Huijie Yan, Yun You, Xinjian Li, Lei Liu, Fengqian Guo, Qiongling Zhang, Dewen Liu, Yan Tong, Shilan Ding, Jinyu Wang

**Affiliations:** Institute of Chinese Materia Medica, China Academy of Chinese Medical Sciences, Beijing, China

**Keywords:** mesoporous silica, active targeted, folic acid, RGD peptide, MCF-7 cells, paclitaxel

## Abstract

**Pharmacological Relevance:**

Paclitaxel (PTX) is currently the only botanical drug that can control the growth of cancer cells. Paclitaxel is widely used in the treatment of breast cancer, ovarian cancer, uterine cancer, non-small cell lung cancer and other cancers.

**Aim:**

Folate receptor and integrin *α*_v_*β*_3_ are highly expressed on the surface of human breast cancer cells MCF-7. Folic acid and arginine-glycine-aspartate (Arg-Gly-Asp, RGD) tripeptide sequence have a high affinity for folate receptor and integrin *α*_v_*β*_3_, respectively. To enhance the effect on breast cancer, we constructed the folate acid and RGD peptide dual-targeted (MSNs-NH_2_-FA-RGD) drug-carrier based on mesoporous silica nanoparticles.

**Methods:**

The structure of mesoporous nanocarriers was characterized by Fourier transform infrared spectroscopy, nitrogen adsorption-desorption analysis, transmission electron microscopy, laser particle size analyzer, and thermogravimetric analysis. Paclitaxel was chosen as the model drug. The targeting-ability was verified by observing the uptake of mesoporous carriers loaded with rhodamine in MCF-7, MCF-10A, and HeLa cells using a fluorescence microscope. The cytotoxicity of the blank carrier MSNs-NH_2_-FA-RGD and the efficacy of the drug carrier PTX@MSNs-NH_2_-FA-RGD were assessed by cell experiments.

**Results:**

The characterization showed successful construction of a dual-targeted mesoporous silica nanocarrier. Obvious differences were detected in the fluorescence intensity of the three cell lines. The results of the pharmacological tests indicated that the blank nanoparticles do not cause any apparent toxicity on these cells. The IC_50_ of free PTX and PTX@MSNs-NH_2_-FA-RGD on MCF-7 cells line treated for 48 h were 35.25±2.57 ng·ml^-1^ and 22.21±3.4 ng·ml^-1^ respectively, which indicated that the inhibitory efficacy of PTX@MSNs-NH_2_-FA-RGD on MCF-7 was 1.6 times than that of free PTX.

**Conclusions:**

The dual-targeted nanocarrier MSNs-NH_2_-FA-RGD could target breast cancer cells, and sever as a potential candidate in future of drug development.

## Introduction

Breast cancer seriously harms women's health. Statistics show that 1.2 million women worldwide suffer from breast cancer each year, and 500,000 women die from breast cancer (Raviv, 2004). In recent years, the patient population has shown a trend of being younger. Traditional chemotherapy exposes many issues, such as poor specificity by chemotherapeutics, drug resistance caused by repeated and large doses of multi-drugs, and side effects to normal tissues. Fortunately, the treatment has received widespread attention in the medical community. Many researchers have put a lot of effort into targeted treatment field. With in-depth research, nanocarriers are found to play an increasingly important role in targeted therapy. Owing to passive or active targeted delivery of drugs, the nanocarriers have shown great potential in improving drug concentration and bioavailability in tumor sites (Darvishi and Farahmand, 2017).

Mesoporous silica nanoparticles (MSNs) are unique among numerous inorganic nanomaterials due to their good biocompatibility, high load capacity, and uniform adjustable pore size (Song et al., 2007; Kazuki et al., 2013). By modifying the surface of MSNs with different substances and groups, the MSNs carrier can be endowed with the ability of targeting and stimulate-responsive, avoiding the early leakage of drugs and increasing the concentration of drugs at the lesion sites. The surface of MSNs could also be easily functionalized with a variety of targeted groups, such as antibody (Zhang et al., 2015; Tao et al., 2016), protein (Pourjavadi and Tehrani, 2016), peptides, and small molecules (Alejandro et al., 2014). Kazuki et al (Kazuki et al., 2013) wrapped the peptide Ac-(VKVS)_4_E-NH_2_ on the surface of MSNs. The conformation of Ac-(VKVS)_4_E-NH_2_ changed in different pH environments, controlling the exposure and coverage of the mesoporous orifice, thereby the carrier system showed pH-dependent release behavior. Xue et al. (2011) utilized the supramolecular force between benzimidazole and β-cyclodextrin, choosing fluorescent dye Hoechst 33342 as model cargo, to construct a cyclodextrin-based silica pH controlled release system. This carrier exhibits an acid-responsive ability to release drugs and induce cancer cell apoptosis in human pancreatic cancer cell PANC-1. Martinez-Carmona et al., (2018) modified the targeted ligand plant lectin concanavalin (ConA) on MSNs to enable the nanocarrier to specifically recognize human osteosarcoma cells. Tian et al., (2016) utilized iron-binding glycoprotein (Tf) not only as an entrant into the target sites but also as a blocking agent to inhibit the release of the drug before entering the tumor cells. He et al., (2012) functionalized MSNs with nucleic acid sequence polyadenylic acid and loaded with coralyne and near-infrared photothermal dye indocyanine green (ICG), constructing a nano-therapy platform combining chemical and photothermal therapy.

**Figure 1:**
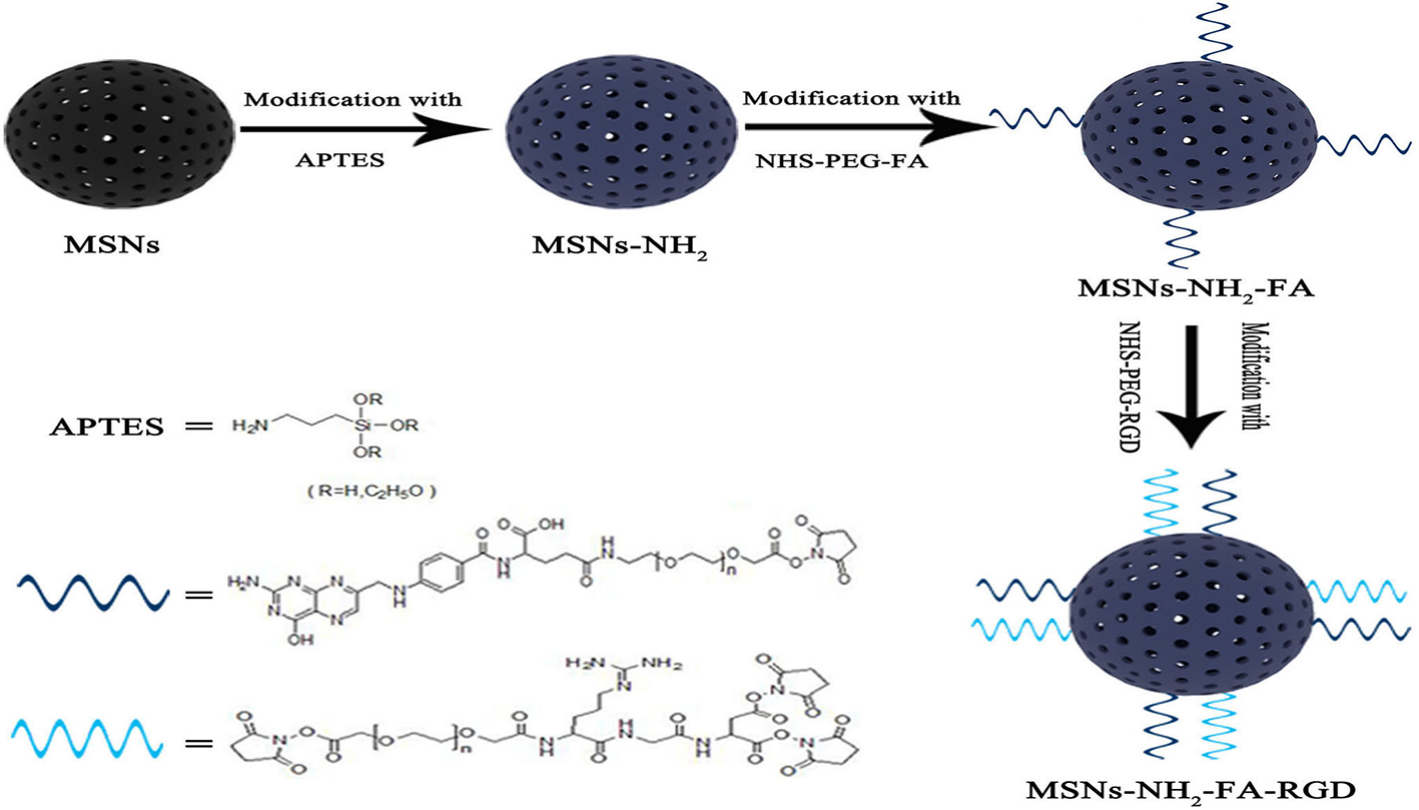
Schematic illustration of preparation process of MSNs-NH_2_-FA-RGD.

Folic acid receptors (FR) and integrin *α*_v_*β*_3_ have the characteristic of expression specificity, that is, they are highly expressed on the surface of tumor cells but not expressed on the surface of normal cells. RGD peptide is a type of short peptide containing arginine-glycine-aspartate (Arg-Gly-Asp, RGD) peptide sequence, which is the smallest unit that can be recognized by integrin *α*_v_*β*_3_ (Hee Dong et al., 2010; Porta et al., 2013). Folic acid (FA) and RGD peptides have strong affinity for FR and integrin *α*_v_*β*_3_, so FA and RGD peptides are often used as targeted modification groups. There have been studies on separately grafting FA or RGD peptide onto mesoporous silica carriers. In this experiment, for the first time, we grafted both FA and RGD peptide on the surface of MSNs as shown in **Figure 1**, constructing a dual-targeting nanocarrier MSNs-NH_2_-FA-RGD. Modifying folic acid groups (NHS-PEG-FA) and RGD peptide groups (NHS-PEG-RGD) onto the surface of MSNs endows the carrier with the ability to actively target cancer sites, and the PEG long chains enhance the *in vivo* stability of the carrier.

Paclitaxel (PTX) is a tetracyclic diterpenoid compound with potent effects on cancers, such as breast cancer, colon cancer, bronchial cancer, and endometrial cancer. PTX could specifically bind to different sites of the tubules and aggregate the microtubules, thereby inhibiting the normal reorganization of the microtubule network and interfering with the cell mitosis. It is one of the most energetic anti-tumor drugs since doxorubicin (Ren et al., 2005). However, PTX has low bioavailability and extremely poor solubility (water solubility of 0.006 g·L^-1^), which brings some difficulties to clinical application (Lv et al., 2014). We chose PTX as a model drug to evaluate the drug loading of MSNs-NH_2_-FA-RGD@PTX. Loading PTX of nanometer-sized into MSNs-NH_2_-FA-RGD would not only solve the problem of poor solubility but also significantly improve bioavailability. FR and integrin *α*_v_*β*_3_ are highly expressed on the surface of MCF-7 cells but not on the surface of human normal breast epithelial cells MCF-10A, and human cervical cancer cells HeLa only express FR on the surface (Shen et al., 2011). Cell experiments were used to evaluate the biocompatibility, anti-cancer efficacy and cellular uptake of nanocarriers.

## Materials and Methods

### Materials

Cetyltrimethylammonium bromide (CTAB), 3-aminopropyltrietho xysilane (APTES), tetraethylorthosilicate (TEOS), and rhodamine (RhB) were purchased from Aladdin Chemical Reagents (Shanghai, China,). Folic acid polyethylene glycol succinimidyl activated ester (FA-PEG-NHS, PEG= 2000) and RGD tripeptide polyethylene glycol succinimidyl activated ester (RGD-PEG-NHS, PEG= 2000) were purchased from Pengshuo Biotechnology Co., Ltd (Shanghai, China). Paclitaxel (purity ≥ 98 %) was purchased from Ruifensi Co., Ltd (Chengdu, China). Lysotracker green DND-22 was obtained from Invitrogen Life Technologies Corporation (Tianjing, China). Other reagents and solvents were provided by Dingguo reagent company (Beijing, China). All the chemical reagents used in this experiment were of analytical grade and used without further purification.

### Characterization

The mesoporous structure and morphology of the nanoparticles were characterized by transmission electron microscopy (TEM) (Tecnai G2 F30, USA) at an accelerated voltage of 300 kV. The dispersing agent is water and the dispersions are stabile in 12 h. Nitrogen adsorption-desorption analysis at 77 K was carried out on an adsorption analyzer (ASAP 2460, Micromeritics, USA). Zeta potential and particle diameter experiments were performed at 25 °C using Malvern ZetaSizer Nano-S90. The Fourier transform infrared (FTIR) spectra were obtained on an FTIR spectrometer (Nexus, Thermo Nicolet, USA). Thermogravimetric analysis (TGA) was performed by a Thermo Gravimetric Analyzer (STA8000, Perkin Elmer, USA) under N_2_ atmosphere at a heating rate of 10 °C·min^-1^. All fluorescence spectra were obtained on a fluorescence microscope (Hitachi F-7000 FL Spectrophotometer).

### Preparation of MSNs-NH_2_-FA-RGD

3 g CTAB was solubilized in 1440 ml of deionized water in three-necked flask heated to 80°C in an oil bath. Then the temperature of the CTAB solution was adjusted to 80°C before adding 10.5 ml sodium hydroxide aqueous solution (2.0 mol·L^-1^), followed by dropwise addition of 15 ml TEOS under vigorous stirring. After 2 h, the resultant product was collected by filtration using a suction pump and rinsed with ethanol. To remove the surfactant template CTAB, the product was calcined at 550°C for 4 h in a muffle furnace to obtain MSNs.

The introduction of aminopropyl groups through the post-grafting process was conducted by dispersing 0.9 g MSNs in 90 ml toluene, followed by the addition of 434 μl APTES. The mixture was refluxed and stirred at 90°C in an oil bath for 6 h, followed by centrifugation with 10000 rpm for 15 min and washing with ethanol and distilled water at room temperature. The resultant product was dried to a constant weight under vacuum to obtain MSNs-NH_2_.

An equivalent of 40 mg of NHS-PEG-FA was dispersed by ultrasonication in 50 ml dimethyl sulfoxide (DMSO), and the pH of the system was adjusted to be alkaline by triethylamine. Then, 200 mg of MSNs-NH_2_ was mixed in DMSO by then magnetic stirring for 4 h. The solids were collected by centrifugation and washing with ethanol. After drying under a vacuum atmosphere, MSNs-NH_2_-FA was obtained. MSNs-NH_2_-FA-RGD was synthesized similarly. 200 mg of MSNs-NH_2_-FA was dispersed in DMSO-triethylamine with 40 mg of NHS-PEG-RGD. After 4 h, the reaction product was collected by centrifugation, washing, and vacuum drying.

### Preparation of Drug-Loaded Nanocarriers

10 mg PTX and 20 mg MSNs-NH_2_-FA-RGD were ultrasonically dispersed in 20 ml absolute ethanol and magnetically stirred for 24 h at room temperature to load the drug. Subsequently, the PTX-loaded MSNs-NH_2_-FA-RGD (denoted as PTX@MSNs-NH_2_-FA-RGD) was collected by suction filtration, with the surface adsorbed PTX washed away by phosphate-buffered saline (PBS) (pH=7.4). PTX@MSNs-NH_2_-FA-RGD were collected after vacuum drying.10 mg drug-loaded particles was placed in a volumetric flask with methanol, followed by sonication for 1 h and analysis by high-performance liquid chromatography (HPLC). Drug loading rate (%) and entrapment rate (%) were calculated by HPLC at maxima wavelength of 229 nm using the following equation which was quoted from the Pharmacopoeia of the People's Republic of China:

$$\text{Drug}\;\text{loading}\;\text{rate}=(W_{1}-W_{2})/W_{nanocarriers}\times 100\%$$

$$\text{Encapsulation}\;\text{rate}=(W_{1}-W_{2})/W_{1}\times 100\%$$

where *W_1_*, *W_2_* and *W_nanocarriers_* represented the weight of PTX added, the weight of PTX in supernatant and the weight of nanocarriers.

### RhB-Labeled Nanocarriers

RhB was used as a guest molecule to evaluate the ability of targeting tumor sites because of its fluorescence properties. 200 mg of MSNs-NH_2_, MSNs-NH_2_-FA, and MSNs-NH_2_-FA-RGD nanoparticles were mixed in the ethanol solution of RhB (0.4 mg·ml^-1^) for 4h. After centrifugation for 15 min at room temperature with 10000 rpm, the solid particles were dried in vacuum to constant weight. The RhB-labeled mesoporous silica nanoparticles were termed as RhB@MSNs-NH_2_, RhB@MSNs-NH_2_-FA, RhB@MSNs-NH_2_-FA-RGD, respectively.

### Cell Culture

The cell culture tests were performed using HeLa, MCF-7, and MCF-10A cells purchased from the American Type Culture Collection (Manassas, VA, USA). MCF-7 cells and HeLa cells were cultured in RPMI 1640 medium with 10% heat-inactivated fetal bovine serum (FBS). MCF-10A cells were cultured in DMEM/F12 medium with 5 % horse serum, 10 μg·ml^-1^ insulin, 20 ng·ml^-1^ EGF, 100 ng·ml^-1^ cholera toxin, and 0.5 μg·ml^-1^ hydrocortisone. All cells were cultivated in an incubator with 5 % CO_2_ at 37°C.

### Cell Uptake and Location

Collect HeLa, MCF-7, and MCF-10A cells in the logarithmic growth phase and seed them in 96 wells at a density of 6 × 10^4^, 6 × 10^4^, and 1.5 × 10^5^ cells/ml. After the cells were incubated for 24 h, aspirate the medium. The cells were incubated with RhB@MSNs-NH_2_, RhB@MSNs-NH_2_-FA, and RhB@MSNs-NH_2_-FA-RGD (20 µg·ml^-1^) for 4 h. Each well was washed three times with cold PBS to remove the nanoparticles not internalized into the cells and then the cell morphology was fixed with 4% paraformaldehyde for 5 min. Subsequently, the nucleus was stained with DAPI for 5 min, while lysosomes were identified using the dye named Lysotracker. Fluorescence microscopy of fluorescein-labeled cells was performed with an Imaging System equipped with three Led Lights Cubes (BioFlux 1000Z, USA, Fluxion Biosciences).

### *In Vitro* Toxicity Test of Blank Carrier

The CCK- 8 method was used to determine the toxicity of the blank nanocarrier MSNs-NH_2_-FA-RGD to MCF-7 cells. Collect MCF-7 cells in the logarithmic growth phase and seed them in 96 wells at a density of 6 × 10^4^ cells/ml. After the cells were incubated for 24 h, we aspirated the medium and added 100 μl of complete medium containing different concentrations of MSNs-NH_2_-FA-RGD (concentrations of 20, 40, 80, and 160 μg·ml^-1^) to each well. Cultivate MCF-7 cells in a constant temperature incubator for 24 h or 48 h. Measure the absorbance of each well at 450 nm by micro plate reader (Thermo scientific, USA) and calculate the inhibition rate.

### *In Vitro* Antitumor Drug Efficacy

Collect MCF-7 cells in the logarithmic growth phase and seed them in 96 wells at 6 × 10^4^ cells/ml. Configure the complete medium for PTX@MSNs-NH_2_-FA-RGD and free PTX to different concentrations (based on the PTX concentration as a quantitative basis, and set the concentration gradient to 10, 30, 100, 300, 1000 ng·ml^-1^). After culturing MCF-7 cells for 24 h, aspirate the medium and add complete medium with different concentrations of PTX mentioned above. Cultivate MCF-7 cells in constant temperature incubator for 24 h and 48 h. Measure the absorbance of each well at 450 nm by a microplate reader (Thermo scientific, USA) and calculate the inhibition rate.

## Results and Discussion

### Preparation and Characterization of MSNs-NH_2_- FA-RGD Nanocarrier

**Figure 2:**
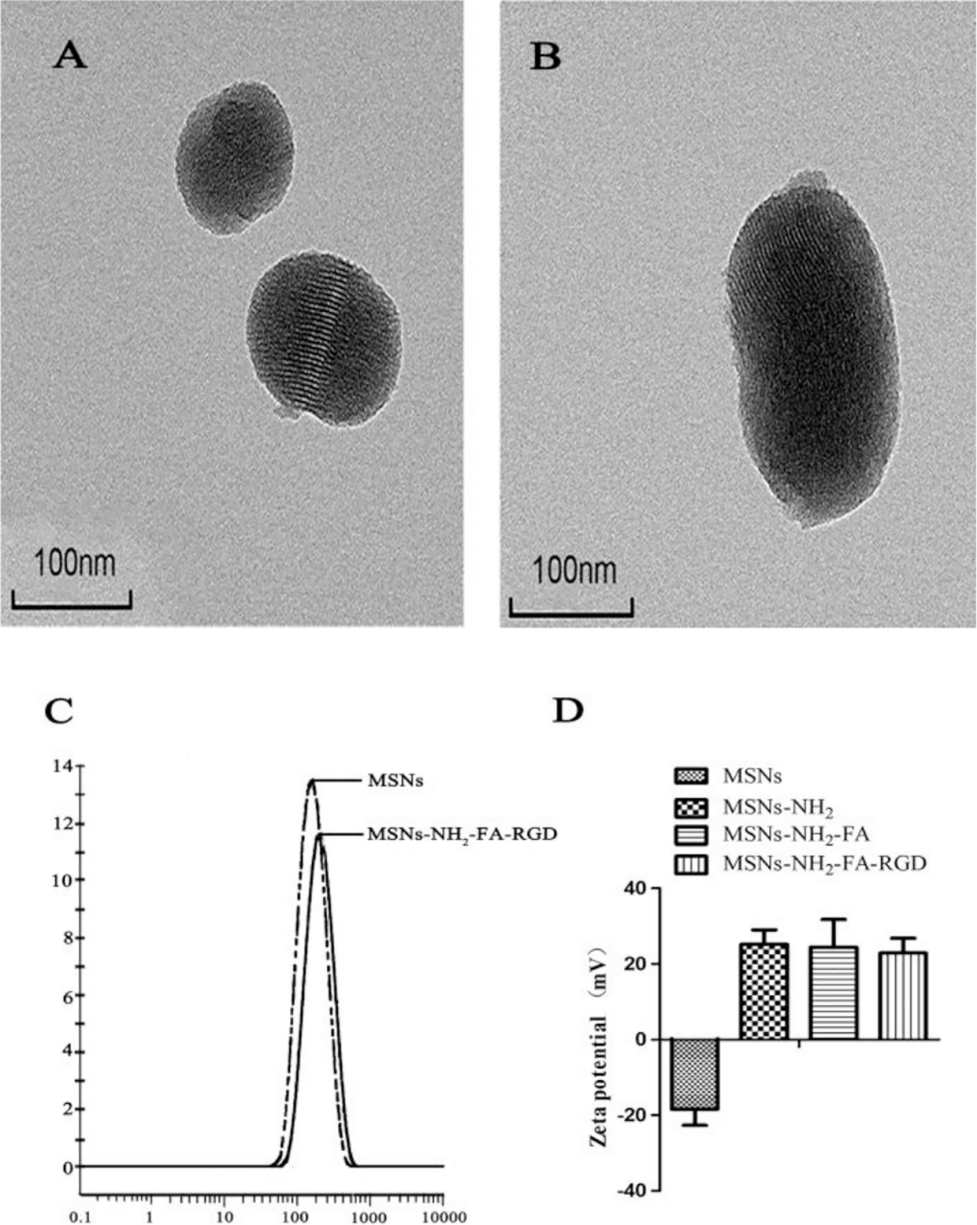
TEM images of **(A)** MSNs and **(B)** MSNs-NH_2_-FA-RGD. **(C)** Particle size distribution of MSNs and MSNs-NH_2_-FA-RGD in deionized water. **(D)** Zeta potential of MSNs, MSNs-NH_2_, MSNs-NH_2_-FA, and MSNs-NH_2_-FA-RGD in deionized water.

TEM images showed that the MSNs and MSNs-NH_2_-FA-RGD nanoparticles were spherical, with smooth surface and even distribution (**Figures 2A, B**). After modification, the ordered mesopores could still be directly observed from **Figure 2B**, which proved that modified process would not affect the mesoporous structure of them. The laser particle size analyzer showed that the average particle sizes of MSNs and MSNs-NH_2_-FA-RGD were 188.6 nm (PDI= 0.267) and 204.1 nm (PDI= 0.269), respectively (**Figure 2C**). Zeta potential of MSNs in distilled water was -18.4 ± 4.30 mV. Because of the amino group on the surface, zeta potential of MSNs-NH_2_ was reversed to 25.6 ± 3.8 mV after the process of amination. Due to the PEG long chains on the targeted group covering the positive charge of MSNs-NH_2_, the positive potential of MSNs-NH_2_-FA, and MSNs-NH_2_-FA-RGD have decreased to 24.4 ± 7.36 mV and 22.9 ± 3.9 mV, respectively (**Figure 2D**).

**Figure 3:**
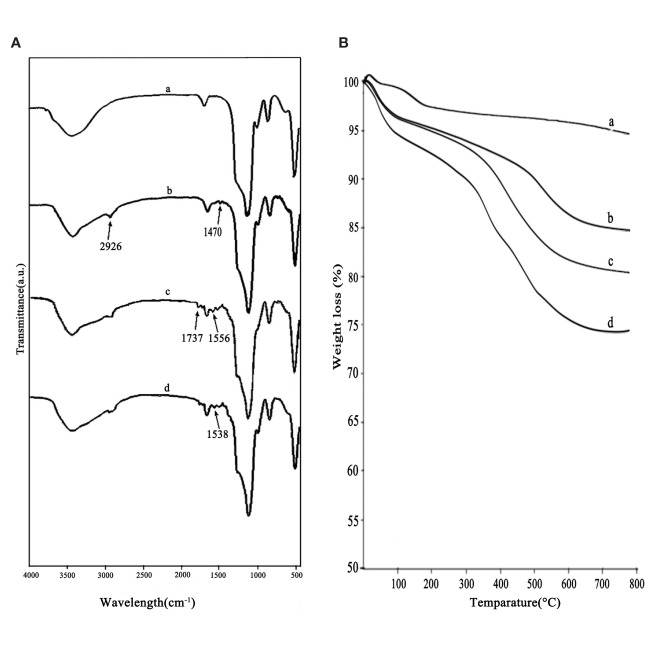
FTIR spectra **(A)** and thermograms **(B)** of MSNs (a), MSNs-NH_2_ (b), MSNs-NH_2_-FA (c), and MSNs-NH_2_-FA-RGD (d).

**Figure 3A** showed the FT-IR spectra of a) MSNs, b) MSNs-NH_2_, c) MSNs-NH_2_-FA, and d) MSNs-NH_2_-FA-RGD. As shown in curve (a), the strongest absorption peak at 1083 cm^-1^ was the symmetric stretching vibration peak of Si-O-Si. 3400 cm^-1^ and 1640 cm^-1^ were the stretching vibration peak and bending vibration peak of Si-OH, respectively, indicating that there were hydroxyl groups with different bonds and states on the surface of SiO_2_. In the curve (b), the stretching vibration peak of the methylene group at 2926 cm^-1^ and the bending vibration peak of the amino group at 1470 cm^-1^ both indicated that the amination process was successful. In the curve (c), the C=O vibration absorption peak and the O=C-N-H absorption peak at 1737 cm^-1^ and 1556 cm^-1^ indicated that FA was grafted on the surface. The new absorption peak at 1538 cm^-1^ in curve (d) was assigned to the amide bond which was affected by the RGD peptide and shifted to the direction of short wave number. **Figure 3B** showed the thermograms of a) MSNs, b) MSNs-NH_2_, c) MSNs-NH_2_-FA, and d) MSNs-NH_2_-FA-RGD. According to analysis of Pyris software, the weight loss from room temperature to 800°C of MSNs, MSNs-NH_2_, MSNs-NH_2_-FA, and MSNs-NH_2_-FA-RGD were 4.4%, 14.51%, 19.04%, and 24.37%, respectively. Weightlessness in different temperature ranges represents different meaning (Pourjavadi and Tehrani, 2014). Take MSNs as an example, the weight loss below 200°C was 2.03 %, causing by absorbed water in the air, and the proportion of this part of weightlessness can be directly read on the graph through the software. The weightlessness between 200°C and 800°C could be attributed to the removal of organic groups. The weightlessness of MSNs above 200°C was 2.37 %, which could be indicated as the incomplete removal of CTAB. After deducting the proportion of CTAB and absorbed water, the weightlessness between 200°C to 800°C of MSNs-NH_2_, MSNs-NH_2_-FA, and MSNs-NH_2_-FA-RGD were 10.30%, 14.17%, and 18.56%, respectively. And the graft ratios of the amino group, FA, and RGD peptide were about 7.93%, 3.87%, and 4.39%.

**Figure 4:**
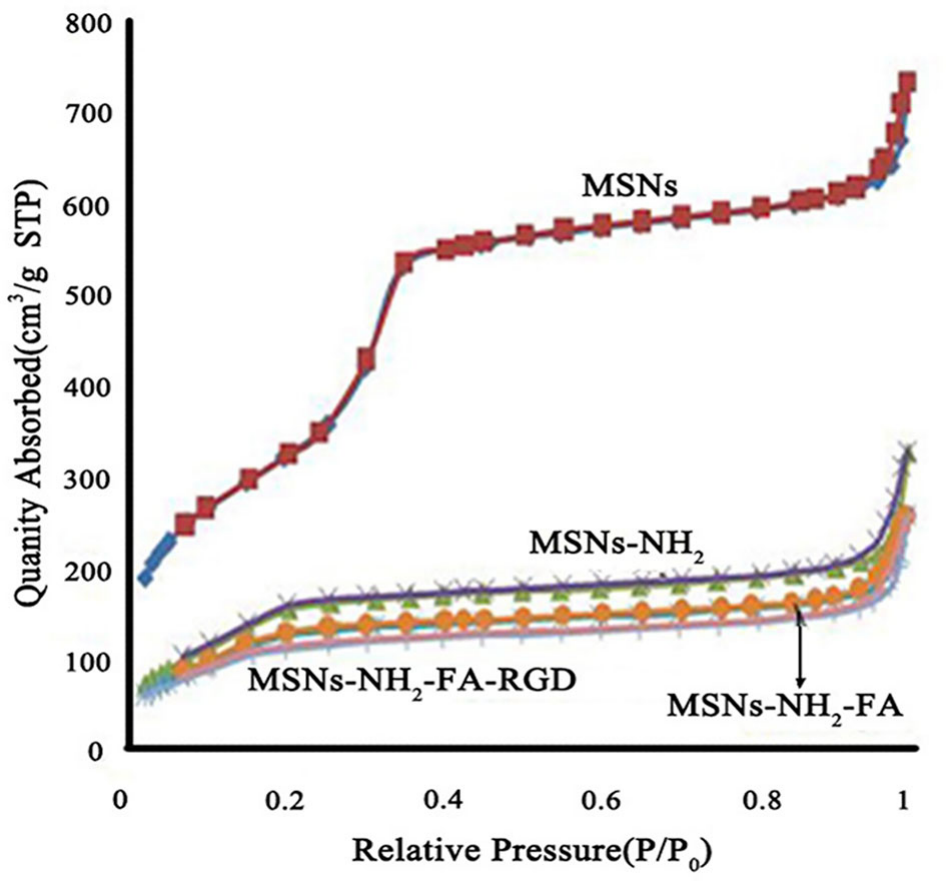
Nitrogen adsorption-desorption isotherms of MSNs, MSNs-NH_2_, MSNs-NH_2_-FA, and MSNs-NH_2_-FA-RGD.

**Figure 4** showed the nitrogen adsorption-desorption isotherms and the corresponding pore size distributions of the MSNs, MSNs-NH_2_, MSNs-NH_2_-FA, and MSNs-NH_2_-FA-RGD. Textural parameters were listed in **Table 1**. It could be observed that the adsorption amount of N_2_ increased slowly when P/P_0_ was less than 0.25 from the adsorption isotherms of MSNs, because the adsorption of N_2_ on the surface of the sample channel occurred in single-molecule and multi-molecular layers. When P/P_0_ was between 0.25-0.4, the adsorption amount of N_2_ raised sharply. A steep platform peak was observed on the adsorption isotherm curve with the reason that N_2_ could cause capillary condensation in the sample channel at low temperature, indicating that there was ordered mesoporous structure and uniform pore size distribution in the MSNs sample. When P/P_0_ was between 0.4-0.95, the curve was relatively flat, due to the adsorption of N_2_ on the outer surface. When P/P_0_ was more than 0.95, the curve appeared a small jump, the reason was that the capillary condensation caused by the pores between the particles. With the introduction of FA and RGD peptide, the relative pressure value P/P_0_ of the steep peak decreased, and the specific surface area, pore volume and pore size of MSNs-NH_2_, MSNs-NH_2_-FA, and MSNs-NH_2_-FA-RGD also decreased, proving that the amino group, NHS-PEG-FA and NHS-PEG-RGD covered on the surface of MSNs.

**Table 1:** The nitrogen adsorption-desorption parameters of different functionalized MSNs samples.

Samples	Surface area (m^2^/g)	Pore size (nm)	Pore volume (cm^3^/g)
MSNs	1203.41	3.77	1.13
MSNs-NH_2_	659.34	3.09	0.51
MSNs-NH_2_-FA	514.86	3.07	0.40
MSNs-NH_2_-FA-RGD	426.68	3.03	0.40

### Drug Loading Efficiency

In order to estimate the ability of drug-loading of MSNs-NH_2_-FA-RGD, PTX was chosen as the model drug. The loading and entrapment efficiency were estimated at 18.7% and 85.2%, respectively.

### *In Vitro* Cytotoxicity of Blank Nanoparticles

**Figure 5:**
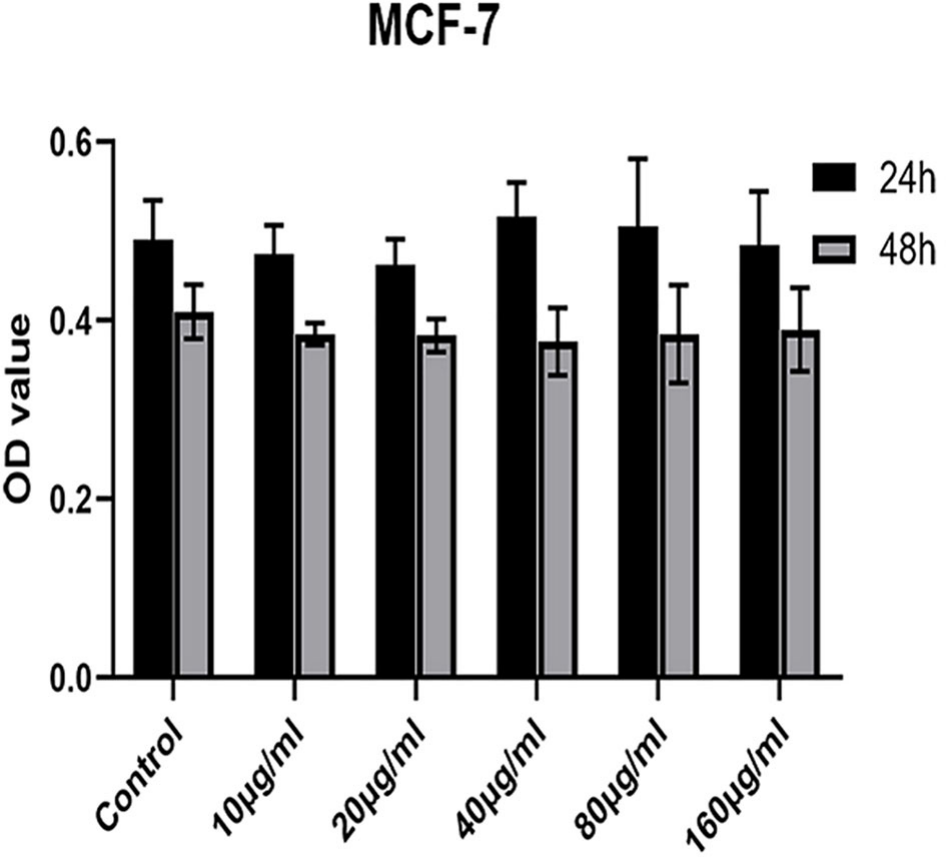
Cell viability of MCF-7 cells incubation with MSNs-NH_2_-FA-RGD at different concentrations (µg·ml^-1^) after 24 h and 48 h.

A safe and effective nanocarrier system is a prerequisite to *in vivo* therapy. Therefore, we explored the *in vitro* cytotoxicity of MSNs-NH_2_-FA-RGD nanoparticles. As shown in **Figure 5**, MSNs-NH_2_-FA-RGD did not show any cytotoxicity compared with the blank control group at any of the concentrations used, even incubated after for 48 h, demonstrating that MSNs-NH_2_-FA-RGD nanoparticles had excellent cytocompatibility.

### Cell Uptake Assay

**Figure 6:**
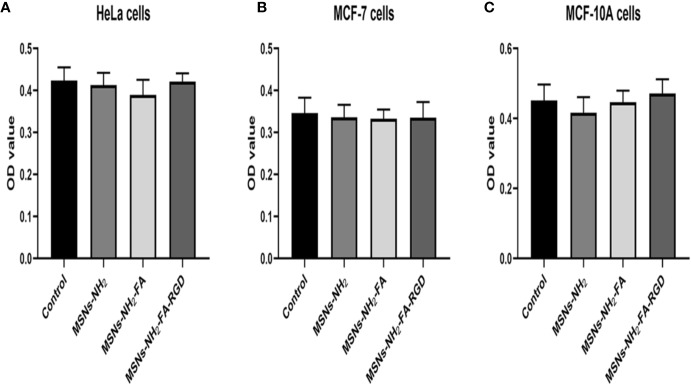
Cell viability of HeLa **(A)**, MCF-7 **(B)** and MCF-10A **(C)** cells after 24 h incubation with MSNs-NH_2_, MSNs-NH_2_-FA and MSNs- NH_2_-FA-RGD at 20 µg·ml^-1^.

Firstly, we evaluated the cytotoxic potential of MSNs-NH_2_, MSNs-NH_2_-FA, and MSNs-NH_2_-FA-RGD at 20 µg·ml^-1^ in HeLa, MCF-7, and MCF-10A cells for 24 h. The above three blank nanoparticles did not exert any apparent toxicity on the viability of these cells from **Figure 6**.

**Figure 7:**
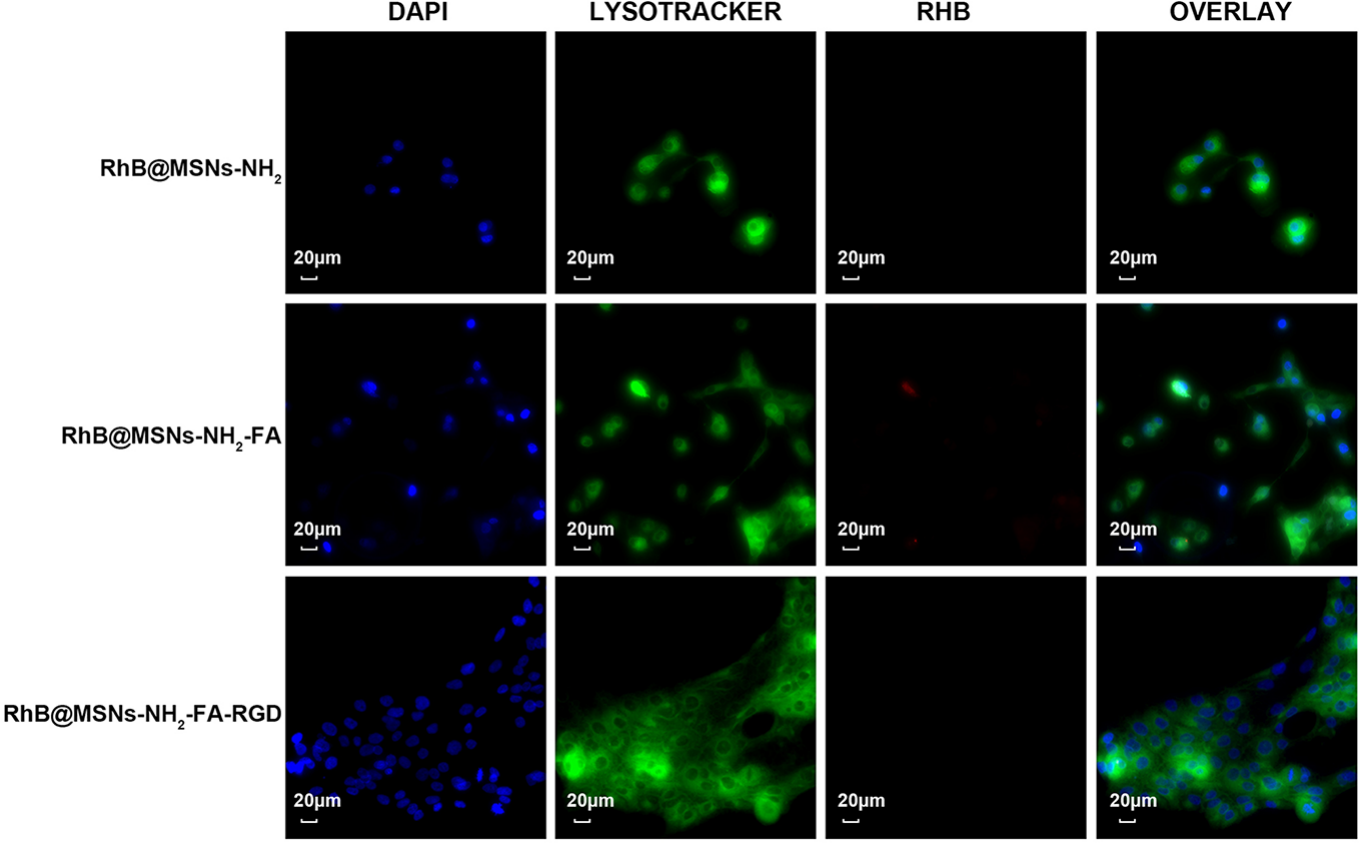
Fluorescence microscopy images of MCF-10A cells incubation with RhB@MSNs-NH_2_, RhB@MSNs-NH_2_-FA, and RhB@MSNs-NH_2_-FA-RGD for 4 h. Blue fluorescence field: nucleus; green fluorescence field: cytoplasm; red fluorescence field: a dye used to label nanocarriers. Scale bar: 20 μm.

**Figure 8:**
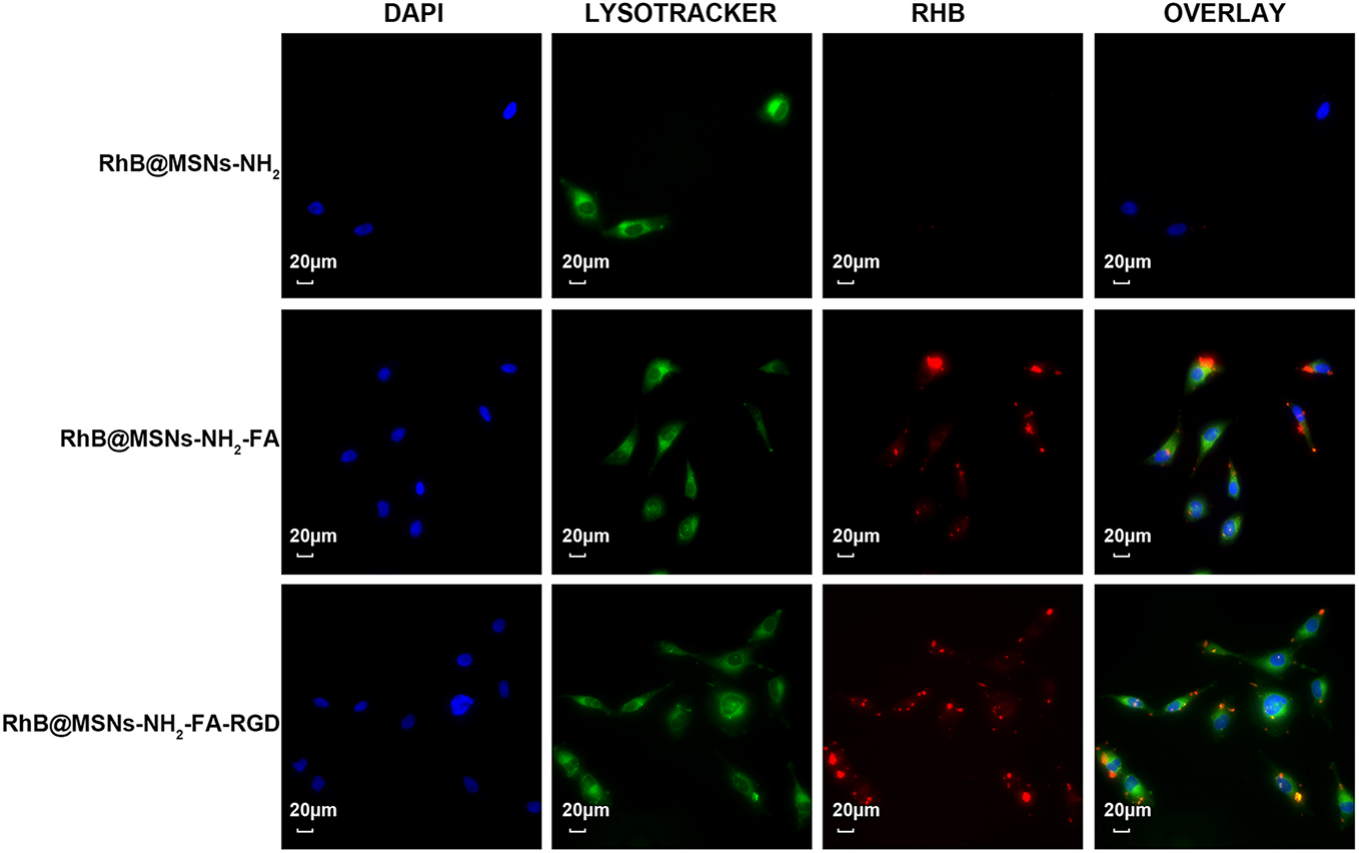
Fluorescence microscopy images of HeLa cells incubation with RhB@MSNs-NH_2_, RhB@MSNs-NH_2_-FA, and RhB@MSNs-NH_2_-FA-RGD for 4 h. Blue fluorescence field: nucleus; green fluorescence field: cytoplasm; red fluorescence field: a dye used to label nanocarriers. Scale bar: 20 μm.

**Figure 9:**
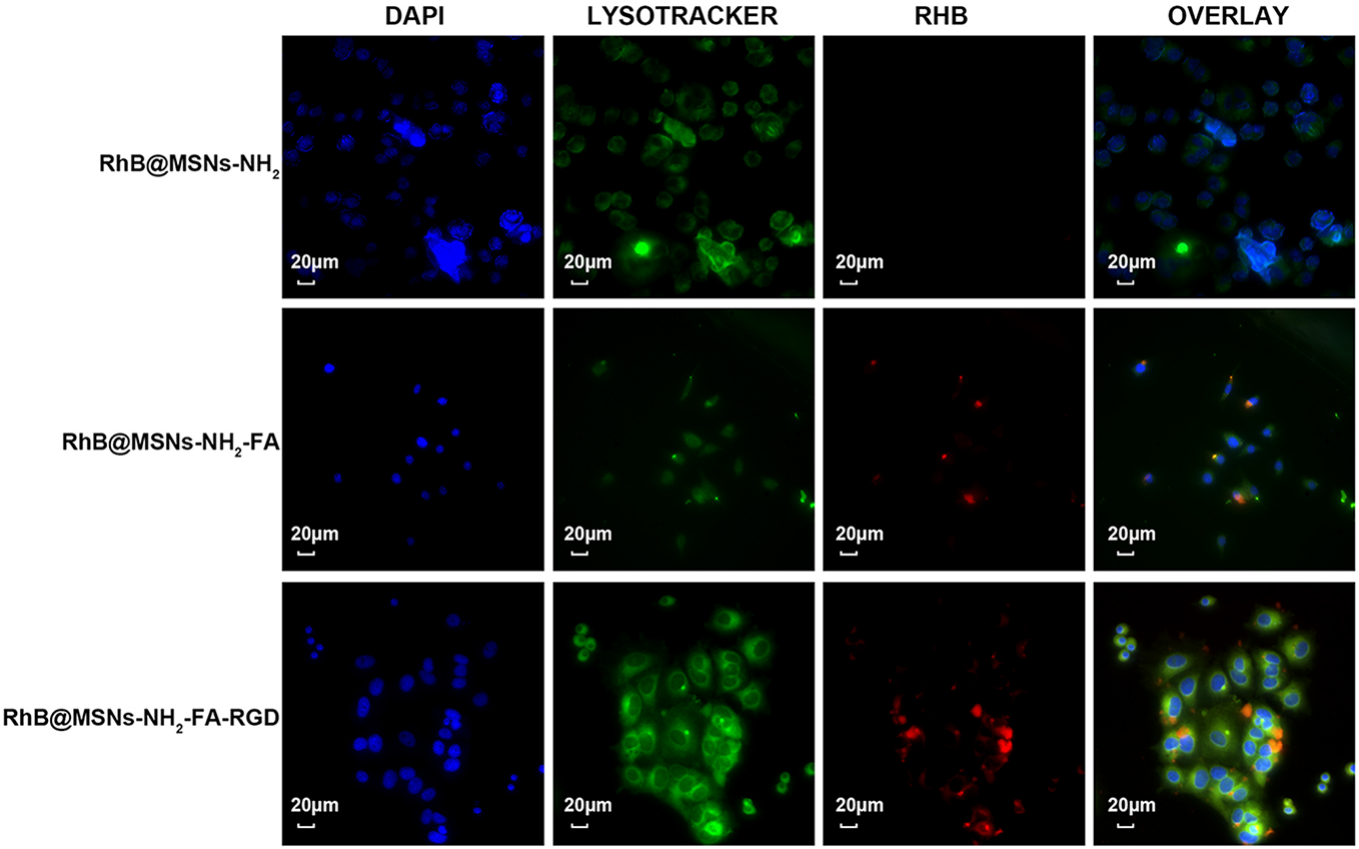
Fluorescence microscopy images of MCF-7 cells incubation with RhB@MSNs-NH_2_, RhB@MSNs-NH_2_-FA, and RhB@MSNs-NH_2_-FA-RGD for 4 h. Blue fluorescence field: nucleus; green fluorescence field: cytoplasm; red fluorescence field: a dye used to label nanocarriers. Scale bar: 20 μm.

As shown in **Figure 7**, the three nanocarriers RhB@MSNs-NH_2_, RhB@MSNs-NH_2_-FA, RhB@MSNs-NH_2_-FA-RGD showed no red RhB fluorescence in MCF-10A cells, indicating that MCF-10A cells hardly took up these three nanocarriers. Because of an excessive amount of FR on the surface, HeLa cells could specifically uptake the nanocarriers modified with FA groups. As shown in **Figure 8**, the red fluorescence representing nanocarriers coincided with the green lysosome region, proving that RhB@MSNs-NH_2_-FA and RhB@MSNs-NH_2_-FA-RGD were endocytosed and distributed in the cytoplasm of HeLa cells, but the fluorescence was not significantly different. There was no obvious fluorescence in the RhB@MSNs-NH_2_ group. **Figure 9** showed that compared with RhB@MSNs-NH_2_, RhB@MSNs-NH_2_-FA, and RhB@MSNs-NH_2_-FA-RGD showed obvious red fluorescence in MCF-7 cells, and RhB@MSNs-NH_2_-FA-RGD had the strongest fluorescence intensity in them. This indicated that targeted modification with FA and RGD peptides enhanced the enrichment of nanocarriers in MCF-7 cells, and the double-targeted effect of modification was better, fully highlighted the advantages of receptor-mediated targeting.

### *In Vitro* Antitumor Drug Efficacy

**Figure 10:**
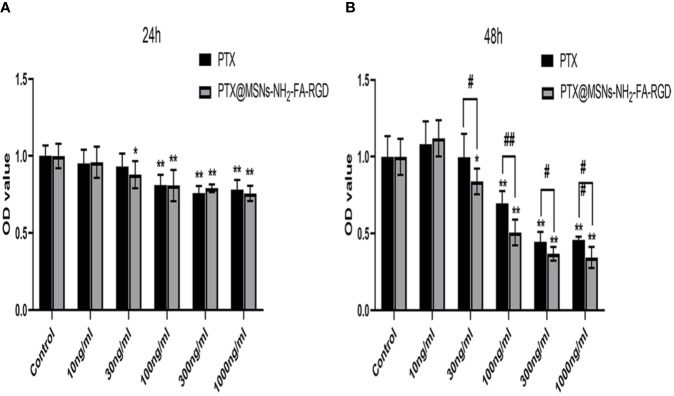
Cytotoxicity for free PTX and PTX@MSNs-NH_2_-FA-RGD at different concentrations (ng·ml^-1^) against MCF-7 cells evaluated at 24 h **(A)** and 48 h **(B)**. (^**^p < 0.01, ^*^p < 0.05 as compared with the data of control group; ^##^*p* < 0.01, ^#^*p* < 0.05 when PTX group compared with PTX@MSNs-NH_2_-FA-RGD at the same concentration).

**Figure 10** showed the inhibition rate of MCF-7 cells by PTX and PTX@MSNs-NH_2_-FA-RGD at different concentrations after 24 h and 48 h. MSNs-NH_2_-FA-RGD had no significant inhibitory effect on MCF-7 cells, therefore the toxic effects of PTX@MSNs-NH_2_-FA-RGD on MCF-7 cells were all from PTX. The experimental results showed that both free PTX and PTX@MSNs-NH_2_-FA-RGD showed inhibitory effects on MCF-7 cells, and the inhibitory effects were concentration-dependent and time-dependent. At 24 h of incubation, the inhibitory effects of PTX@MSNs-NH_2_-FA-RGD and free PTX on MCF-7 cells did not show significant difference (P> 0.05). But after 48 h, it could be clearly seen that PTX@MSNs-NH_2_-FA-RGD had stronger inhibitory effect on MCF-7 cells. The IC_50_ of free PTX and PTX@MSNs-NH_2_-FA-RGD on MCF-7 cells line treated for 48 h were 35.25 ± 2.57 ng·ml^-1^ and 22.21 ± 3.4 ng·ml^-1^, respectively, which indicated that the inhibitory efficacy of PTX@MSNs-NH_2_-FA-RGD on MCF-7 was 1.6 times than that of free PTX.

## Conclusion

Based on the verification of a series of chemical and cell experiments, we synthesized FA and RGD dual-targeted nanocarrier MSNs-NH_2_-FA-RGD. RhB@MSNs-NH_2_-FA-RGD exhibited excellent fluorescence property *in vitro*. The fluorescence microscopy experiment illustrated that RhB@MSNs-NH_2_-FA-RGD had a higher cellular uptake by MCF-7 cells and HeLa cells than MCF-10A cells via receptor-mediated endocytosis. In addition, The IC_50_ of free PTX and PTX@MSNs-NH_2_-FA-RGD for 48h were 35.25 ± 2.57 ng·ml^-1^ and 22.21 ± 3.4 ng·ml^-1^, respectively. The killing capacity of PTX@MSNs-NH_2_-FA-RGD to MCF-7 cells was 1.6 times than that of free PTX, indicating that PTX@MSNs-NH_2_-FA-RGD had higher antitumor activity. The animal experiments are under going. These results indicated that MSNs-NH_2_-FA-RGD could target breast cancer cells, and sever as a potential candidate in future of drug development.

## Data Availability Statement

The raw data supporting the conclusions of this article will be made available by the authors, without undue reservation.

## Author Contributions

HY participated in the literature search, study design, surgery operation, data collection, data analysis, data interpretation, and writing of the manuscript. YY and XL carried out the data collection and analysis and provided the critical revision of the manuscript. LL, FG, QZ, and YT conceived the study and participated in its design and coordination. All authors read and approved the final manuscript. JW and SD participated in the study design and provided critical revision. All authors contributed to the article and approved the submitted version.

## Funding

The work was supported by the Major National Science and Technology Program of China for Innovative Drug (2017ZX09101002-001-001) and the Fundamental Research Funds for the Central public welfare research institutes (ZXKT17025).

## Conflict of Interest

The authors declare that the research was conducted in the absence of any commercial or financial relationships that could be construed as a potential conflict of interest.
